# MCT1 relieves osimertinib-induced CRC suppression by promoting autophagy through the LKB1/AMPK signaling

**DOI:** 10.1038/s41419-019-1844-2

**Published:** 2019-08-13

**Authors:** Ping Jin, Jingwen Jiang, Na Xie, Li Zhou, Zhao Huang, Lu Zhang, Siyuan Qin, Shuyue Fu, Liyuan Peng, Wei Gao, Bowen Li, Yunlong Lei, Edouard C. Nice, Changlong Li, Jichun Shao, Ke Xie

**Affiliations:** 1 State Key Laboratory of Biotherapy and Cancer Center, West China Hospital, and West China School of Basic Medical Sciences & Forensic Medicine, Sichuan University, and Collaborative Innovation Center for Biotherapy, Chengdu, 610041 P. R. China; 2 Department of Biochemistry and Molecular Biology, Molecular Medicine and Cancer Research Center, Chongqing Medical University, Chongqing, 400016 P. R. China; 3 Department of Biochemistry and Molecular Biology, Monash University, Clayton, VIC 3800 Australia; 4 West China School of Basic Medical Sciences & Forensic Medicine, Sichuan University, Chengdu, 610041 China; 5 Department of Urology, Second Affiliated Hospital of Chengdu Medical College (China National Nuclear Corporation 416 Hospital), Chengdu, Sichuan China; 6 Department of Oncology, Sichuan Academy of Medical Sciences and Sichuan Provincial People’s Hospital, School of Medicine, University of Electronic Science and Technology of China, Chengdu, Sichuan 610054 P. R. China

**Keywords:** Cancer, Cell biology

## Abstract

Colorectal cancer (CRC) is one of the most frequently diagnosed cancers worldwide. Development of novel chemotherapeutics is still required to enable successful treatment and improve survival for CRC patients. Here, we found that osimertinib (OSI) exhibits potent anti-CRC effects by inducing apoptosis, independent of its selective inhibitory activity targeting the EGFR T790M mutation. Intriguingly, OSI treatment triggers autophagic flux in CRC cells. Inhibition of autophagy markedly augments OSI-induced apoptosis and growth inhibition in CRC cells, suggesting a protective role of autophagy in response to OSI treatment. Mechanistically, OSI upregulates the expression of monocarboxylate transporter 1 (MCT1) and subsequently activates LKB1/AMPK signaling, leading to autophagy induction in CRC cells. Notably, OSI significantly exaggerates the sensitivity of CRC cells to the first-line drugs 5-fluorouracil or oxaliplatin. Taken together, our study unravels a novel mechanism of OSI-mediated protective autophagy involving MCT1/LKB1/AMPK signaling, and suggests the use of OSI as a potential agent for clinical CRC treatment.

## Introduction

Colorectal cancer (CRC) is the third most frequently diagnosed cancer and one of the leading causes of cancer-related deaths worldwide^1^. The standard treatment for CRC is surgery plus systematic chemotherapy regimens with or without radiation, which has shown favorable therapeutic efficacy and significantly improved the survival of CRC patients^2,3^. However, the prognosis for patients with advanced CRC still remains poor. In addition, a substantial proportion of advanced CRC patients treated with the first-line chemotherapeutic drugs (including 5-FU and oxaliplatin) often relapse due to drug resistance^4^. Thus, there is an urgent need to develop novel potential therapeutic agents for CRC treatment.

Autophagy is a highly conserved process during which de novo-formed membrane-enclosed vesicles engulf aggregated proteins or damaged organelles, and subsequently fuse with lysosomes for degradation^5^. This degradation process enables tumor cells to adapt to multiple cellular stimuli such as nutrient deprivation, oxidative stress and genotoxic stress^6^. Notably, cancer cells express high levels of transcriptional factor such as Mit/TFE and c-Myc in the nucleus to maintain persistent activation of autophagy for cell survival^7–9^. Thus, inhibition of autophagy could be beneficial for cancer therapy as it has already been documented in CRC treatment^10^. However, it has also been demonstrated that autophagy can promote cell death either alone or in association with apoptosis. For example, apoptosis and autophagy induced by dihydrotanshinone could synchronously contribute to the anti-CRC activity through enhanced generation of ROS and caspase-dependent signaling pathways^10,11^. Therefore, determining the functional roles of autophagy in a distinct context is critical for the development of rational autophagy manipulation strategies for successful chemotherapy.

Monocarboxylate transporter 1 (MCT1) is one of the most well studied members of monocarboxylate transporter family that promotes the passive transport of lactate. It plays an important role in metabolic reprogramming and has been observed to be overexpressed in various types of tumors that preferentially utilize lactate for oxidative metabolism^12^, including hepatocellular carcinoma (HCC)^13^, and gastric cancer^14^. In addition, MCT1 was reported to be involved in the resistance of CRC cells to 5-FU, but the underlying mechanism remains largely unknown^15^. Although a link between autophagy and MCT1 upregulation has been observed in HCC cells^16^, whether autophagy, emerging as a mechanism of drug resistance in cancer chemotherapy, plays a role in MCT1-mediated drug resistance remains unclear.

Osimertinib (OSI) is a mutant-selective EGFR inhibitor for the treatment of non-small cell lung cancer (NSCLC) harboring the T790M mutation, exhibiting effective antitumor activity^17^. However, the anti-cancer efficacy of OSI through an EGFR-independent mechanism has rarely been explored. Here, we found that OSI significantly inhibits the growth of CRC cells by stimulating apoptosis irrelevant of its activity targeting the EGFR T790M mutation. Interestingly, OSI upregulates MCT1 and sequentially induces autophagy flux, which in turn ameliorates OSI-induced apoptosis and growth inhibition of CRC cells. Our findings demonstrate a novel role of MCT1 in autophagy modulation, and provide the molecular basis for the use of OSI as a potential therapeutic agent for CRC treatment.

## Materials and methods

### Cell culture and reagents

Human cancer cell lines DLD-1, HT29, HCT116, SW620, LoVo, RKO, SW480 and noncancerous colorectal cell line NCM460 were purchased from the ATCC and maintained in DMEM supplemented with 10% fetal bovine serum (Gibco), in a humidified incubator at 37 °C under 5% CO_2_ atmosphere.

Reagents used in this study were as follows: osimertinib (HY-15772), 3-methyladenine (HY-19312), 5-fluorouracil (HY-90006), oxaliplatin (HY-17371) were purchased from MedChemExpress (MCE). Crystal violet (C0775) and chloroquine diphosphate salt (C6628) were purchased from Sigma. OSI, 5-fluorouracil, oxaliplatin were dissolved in DMSO. 3-methyladenine, crystal violet, and chloroquine diphosphate salt were dissolved in phosphate-buffered saline (PBS).

The following antibodies were used in this study: Cleaved-caspase 3 (Cell Signaling Technology, 9664S), caspase 3 antibody (Cell Signaling Technology, #9662), Atg5 (Cell Signaling Technology, 12994S), Beclin1 (Cell Signaling Technology, 3738), Bcl-2 (Cell Signaling Technology, 15071), PARP (Abcam, ab74290), cleaved-PARP (Abcam, ab32064), Ki67 (Abcam, ab66155), anti-PRKAA/AMPKα (Abcam), anti-phospho-PRKAA/AMPKα (Thr172) (Abcam), anti-MCT1 (Santa Cruz, sc-365501), β-actin (Santa Cruz, sc-1616), LC3 (Novus, NB100-2220), horseradish peroxidase (HRP)-conjugated anti-rabbit secondary antibody (Santa Cruz, sc-2004), horseradish peroxidase (HRP)-conjugated anti-mouse secondary antibody (Santa Cruz, sc-2005). For immunofluorescence, following antibodies were used: goat anti-mouse Alexa Fluor 594, goat anti-rabbit Alexa Fluor 488.

### Colony formation assay

Cells were seeded in 24-well plates for about 3 days, treated with indicated concentrations of OSI for 48 h, and maintained in DMEM for another 10 days. Cells were then fixed with 4% paraformaldehyde in PBS for 30 min and stained with crystal violet. After washing with ddH_2_O for three times, samples were dissolved in 0.1% SDS. The absorbance was then detected at 490 nm.

### EdU labeling assay

The EdU labeling assay was performed in 96-well plate using the EdU Cell Proliferation Assay Kit (Ribobio). After 24 h treatment, 10 μmol/L EdU was added to each well, and the cells were incubated for another 24 h at 37 °C. Cells were then fixed with 4% paraformaldehyde in PBS and stained with reaction cocktail. DAPI was subsequently used for nuclear staining, followed by imaging with DM2500 fluorescence microscope (Leica).

### TUNEL assays and flow cytometry

Cells were plated on glass coverslips in 24-well plates, then treated as indicated for 24 h. DeadEnd^TM^ Fluorometric TUNEL system was used to stain cells after fixation in 4% paraformaldehyde according to the manufacturer’s instructions (Promega, G3250). The apoptotic and nonapoptotic signals were photographed using DM2500 fluorescence microscope (Leica) and then the percentage of cells with DNA nick end-labeling was evaluated.

Annexin V-FITC/PI Detection Kit (KeyGEN BioTECH, KGA108) was used to measure the ratio of apoptotic cells according to the manufacturer’s protocol. Cells were harvested and washed twice with PBS, and then resuspended in 500 μl binding buffer. Annexin V-FITC and PI were added into the cell suspension, respectively, for apoptosis analysis using FACSCalibur flow cytometer (BD FACScan Flow cytometer, United States) and data were analyzed using FlowJo software.

### Caspase 3/7 assay

A caspase3/7 test kit (ATT Bioquest) was used to evaluate apoptosis induced by OSI. Cells were seeded in 96-well plates (20,000 cells/well/90 µl, medium alone was used as the control). The cells were then treated with indicated concentrations of OSI for 24 h. Caspase 3/7 assay loading solution was then added and incubated at room temperature for at least 2 h. The fluorescence intensity was detected at Ex/Em = 350/450 nm using a microplate reader.

### Immunoblotting and immunoprecipitation

Immunoblotting analysis was performed as described previously^18^. For immunoprecipitation, cells were lysed with IP lysis buffer (20 mM Tris, 137 mM NaCl, 10% glycerol, 1% NP-40 and 2 mM EDTA, pH = 8) and subjected to rotation overnight at 4 °C with indicated antibodies (1 μg). Protein A-Sepharose beads (40 μl, GE Healthcare) were then added and incubated with the lysates for 3 h. After centrifugation at 800 × *g*, the beads were washed four times, boiled with loading buffer and analyzed by immunoblotting with the indicated antibodies.

### Immunofluorescence

Cells were plated on the glass coverslips in 24-well plates. After treatment, cells were fixed with 4% paraformaldehyde in PBS for 30 min. After washing with PBS, cells were permeabilized with 0.4% Triton X-100 and blocked with 5% goat serum for 30 min. Indicated primary antibodies were used to incubate with the cells overnight at 4 °C, followed by secondary antibody (DyLight 488–conjugated goat anti-rabbit IgG or DyLight 594–conjugated goat anti-mouse IgG) at 37 °C for 1 h. After staining nuclei with DAPI for 10 min, images were viewed with a confocal laser scanning microscopy (Zeiss). For DQ-BSA assay, cells were pre-probed with DQ-BSA Red (10 μg/ml) for 1 h and then treated with OSI for 24 h. After fixing with 4% paraformaldehyde in PBS for 30 min, images were visualized using a confocal laser scanning microscopy (Carl Zeiss Microimaging).

### RNA interference

Atg5, MCT1, and scramble siRNA were synthesized by Genepharma. The sequences of siRNA were as follows: human Atg5 siRNA, 5′-GCAACUCUGGAUGGGAUU GTT-3′; human MCT1 siRNA pool: 5′-AAGAGGCUGACUUUUCCAAAU-3′, 5′-GACCAUGAUUGGCAAGUAUUU-3′; human LKB1 siRNA pool: 5′-GAAGAAGGAAATTCAACTA-3′ and 5′-CCGUCAAGAUCCUCAAGAAT-3′. The siRNA was transfected with lipofectamine 3000 reagent (Invitrogen) for 48 h according to the manufacturer’s protocol.

### Immunohistochemistry

Tumor xenografts were formalin-fixed, paraffin-embedded, and sectioned in pre-adhered slides. The tissue sections were deparaffinized, rehydrated and blocked with 3% H_2_O_2_ followed by microwave antigen retrieval in citrate buffer (pH 6.0). After blocking with 10% serum for 30 min at 37 °C, tissues were incubated with primary antibody at 4 °C overnight. The tissue sections were incubated with secondary antibody, and then developed with DAB chromogen according to the protocol. All samples were visualized using a DM2500 fluorescence microscope (Leica). Quantitative scoring analysis were performed by multiplying the percentage of staining-positive cells (A: 0, < 5%; 1, 6–25%; 2, 26–50%; 3, 51–75%; 4, >75%) by the intensity (B: 0, negative; 1, weakly positive; 2, positive; 3, strongly positive). The final score for each slide was calculated as A*B.

### Tumor xenograft model

All animal experiments were approved by the Institutional Animal Care and Treatment Committee of Sichuan University. Six-week-old nude mice (BALB/c, 18–20 g each) were purchased from HFK Bioscience Co., Ltd (Beijing). The mice were housed under standard conditions. For the subcutaneous xenograft model, DLD-1 cells (1 × 10^7^ cells/mouse) were suspended in PBS and subcutaneously implanted into flanks of mice. When the tumor volume reached ~200 mm^3^ (14 days post-injection), the mice were divided into a control group (daily intraperitoneal injection of vehicle) and OSI group (daily intraperitoneal injection of 15 mg/kg OSI). The tumor volumes were measured every day and evaluated according to the following formula: tumor volume (mm^3^) = (length × width^2^)/2. The mice were euthanized after two weeks and tumors were harvested.

### Statistical analysis

All statistical analysis and graphics were performed using GraphPad 6 software. One-way ANOVA or Students *t*-test was used to analyze statistical differences. All data are presented as the mean ± SD from at least three individual experiments. A value of *P* *<* 0.05 was considered as statistically significant.

## Results

### OSI inhibits CRC cells growth by stimulating apoptosis in vitro and in vivo

Aberrant amplification of epidermal growth factor receptor (EGFR) plays a key role in the tumorigenesis and progression of CRC^19^, thus represents a potential target for CRC therapy. In this regard, we screened a small-molecule library of 15 EGFR inhibitors and identified OSI as the most effective candidate in suppressing CRC cell growth (Supplementary Fig. 1A, B). Notably, the anti-CRC effect of OSI is independent of the *KRAS* status in CRC cells (Supplementary Fig. 1C, D). In addition, OSI exhibits greater efficacy than olmutinib, another clinical used third-generation EGFR inhibitor, suggesting an EGFR-independent mechanism of OSI in CRC suppression.

**Fig. 1:**
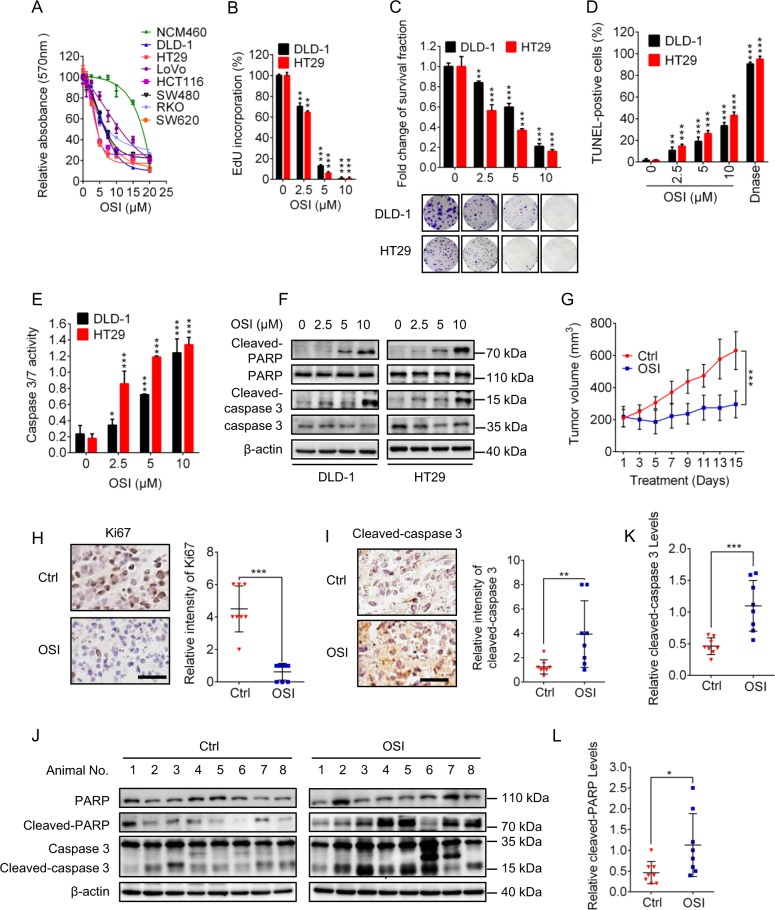
OSI inhibits the growth of CRC cells in vitro and in vivo. **a** The relative absorbance at 570 nm of CRC cell lines (DLD-1, HT29, LoVo, HCT116, SW480, RKO) and noncancerous colorectal cell line NCM460 treated with indicated concentrations of OSI for 24 h. **b**, **c** CRC cells were treated with indicated concentrations of OSI for 24 h. Cell proliferation was detected by EdU incorporation assay (**b**) and colony formation assay (**c**). **d**–**f** In situ TUNEL assay (**d**), caspase 3/7 activity assay (**e**), cleaved-PARP and cleaved-caspase 3 detection (**f**) were used to examine the apoptotic effects in OSI-treated CRC cells. **g** The volume of tumors of mice in cohorts treated with vehicle (*n* = 8) or OSI (*n* = 8) was measured at the indicated time points. **h** The Ki67 expression of tumors was detected by IHC. Scale bar, 50 μm (Left). **i** The Ki67 expression of tumors was detected by IHC. Scale bar, 50 μm (Left). **j** Immunoblotting analysis of cleaved-caspase 3 levels in tumor xenografts obtained from vehicle- or OSI-treated mice. (Each protein of interest from each group was electrophoretically transferred onto a PVDF membrane, incubated with indicated primary and secondary antibodies, and developed as a digital image.) **k** Quantification of cleaved-caspase 3 level in (**j**). **l** Quantification of cleaved-PARP level in (**j**) **P *<* 0.01; ***P < 0.001

To validate the anti-CRC activity of OSI, we detected the relative absorbance of various CRC cell lines (including DLD-1, HT29, LoVo, HCT116, SW480, RKO, and SW620) and noncancerous colorectal cell line (NCM460) in MTT assays following OSI treatment. Indeed, OSI treatment for 24 h markedly decreased the relative absorbance of CRC cell lines, but exhibited less toxicity to noncancerous cells (Fig. 1a). In agreement with these observations, OSI significantly inhibited the proliferation of CRC cells as evidenced by decreased EdU incorporation (Fig. 1b) and colony formation (Fig. 1c). These observations suggest that OSI shows significant anticancer activity against CRC. Further studies described in this work were performed using DLD-1 and HT29 cell lines as they showed the strongest sensitivity to OSI treatment. Since apoptosis is a major form of cell death induced by chemotherapeutic agents^20,21^, we next examined whether OSI could induce apoptosis in CRC cells by analyzing TUNEL-positive cells and caspase3/7 activity. As showed in Fig. 1d, e, OSI-treated CRC cells exhibited obvious induction of apoptosis compared to cells treated with vehicle. This was further confirmed by the increased level of cleaved-PARP and cleaved-caspase 3 in OSI-treated CRC cells (Fig. 1f).

To evaluate the anti-CRC effect of OSI in vivo, a CRC xenograft model was generated by subcutaneously inoculating DLD-1 cells into nude mice. As shown in Fig. 1g, and supplementary Fig. 2A, B, the size, weight and growth rate of tumors were remarkably decreased in OSI-treated mice compared with those of the control group. Furthermore, OSI treatment resulted in relatively weaker Ki67 staining compared with the control group (Fig. 1h). In addition, obvious apoptosis was observed in tumors from OSI-treated mice as evidenced by increased cleaved-caspase 3 intensity and protein levels, as well as upregulated cleaved-PARP levels (Fig. 1j–l). H&E staining of major organs and analysis of body weight changes showed no obvious toxic effect in response to OSI treatment in mice (Supplementary Fig. 2C, D). Collectively, these results demonstrate that OSI exhibits significant antitumor effect in CRC cells by triggering apoptosis both in vitro and in vivo.

### OSI stimulates autophagic flux in CRC cells

**Fig. 2:**
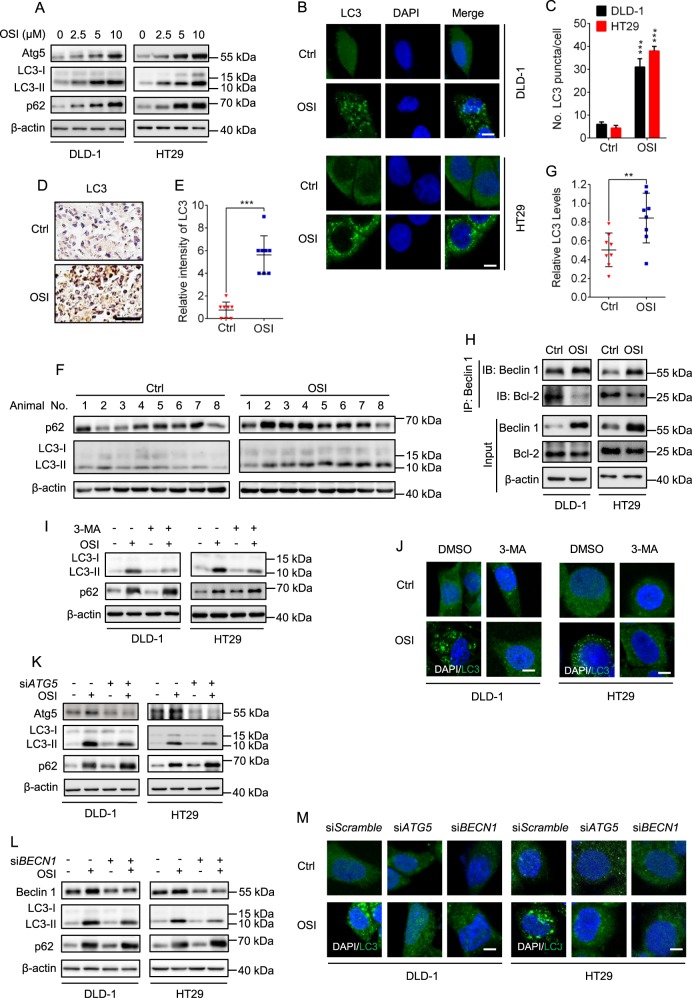
OSI induces autophagy in CRC cells in vitro and in vivo. **a** Immunoblotting analysis of LC3, Atg5, and p62/SQSTM1 expression in CRC cells treated with indicated concentrations of OSI for 24 h. **b** The formation of endogenous LC3 puncta in cells treated with DMSO or 5 μM OSI for 24 h. **c** Total number of endogenous LC3 puncta per cell in (**b**). **d**, **e** LC3 expression in xenograft tissues was examined by IHC. Representative images were provided as indicated in (**d**) and relative intensity of LC3 staining was quantified in (**e**). **f** Immunoblotting analysis of LC3 and p62/SQSTM1 expression in tumor xenografts (Each protein of interest from each group was electrophoretically transferred onto a PVDF membrane, incubated with indicated primary and secondary antibodies, and developed as a digital image.) **g** Relative intensity of LC3 in (**f**). **h** Co-immunoprecipitation analysis of the interaction between Beclin 1 and Bcl-2 in CRC cells treated with or without 5 μM OSI for 24 h. **i** Immunoblotting analysis of LC3 expression in CRC cells treated with or without 5 μM OSI in the presence or absence of 5 mM 3-MA for 24 h. **j** CRC cells were treated as in (**i**), the LC3 puncta were analyzed by immunofluorescence. Scale bar, 10 μm. **k**, **l** Immunoblotting analysis of LC3 expression in CRC cells transfected with si*Scramble*, si*ATG5* (**k**), or si*BECN1* (**l**) for 24 h, followed by treatment with or without 5 μM OSI for another 24 h. **m** CRC cells were treated as in (**k**, **l**). The LC3 puncta were analyzed by immunofluorescence. Scale bar, 10 μm. Data are presented as mean SEM, Student’s *t*-test, and are representative of 3 independent experiments. **P *<* 0.01; ***P *<* 0.001

As autophagy plays an important role in chemotherapy^22^, it is of particular interest to investigate whether autophagy was involved in the anti-CRC effect of OSI. We first evaluated the formation of autophagosomes in OSI-treated cells by analyzing the turnover of LC3-I to lipidated LC3-II and the formation of LC3 puncta, two hallmarks of autophagy^23,24^. OSI treatment resulted in increased LC3-II conversion (Fig. 2a, Supplementary Fig. 3A, B) and LC3 puncta accumulation (Fig. 2b, c, Supplementary Fig. 3C, D). In addition, OSI treatment upregulated the protein levels of Atg5, a key component in the ubiquitin-like conjugating system, in a dose-dependent manner (Fig. 2a). Moreover, stronger LC3 staining (Fig. 2d, e) and increased LC3-II protein levels (Fig. 2f, g) were observed in OSI-treated tumor xenografts. These results indicate that OSI promotes autophagosome accumulation in vitro and in vivo.

The accumulation of autophagosomes may be attributed to either autophagy initiation or blockage of autophagic flux^25^. We thus set out to examine the interaction of Beclin1 with its negative regulator Bcl-2 to validate whether OSI treatment could promotes autophagy initiation^5,23^. As shown in Fig. 2h, OSI treatment disrupted the interaction between Beclin1 and Bcl-2. Furthermore, inhibition of class III PI3K by 3-MA^23^, or siRNA-mediated *ATG5* or *BECN1* silencing, prominently inhibited the conversion of LC3-II and the formation of LC3 puncta in OSI-treated CRC cells (Fig. 2i–m, Supplementary Fig. 3E, F). Taken together, these data indicate that OSI induces autophagosome formation by promoting autophagy initiation in CRC cells. Interestingly, we found that olmutinib, another third-generation EGFR inhibitor, shows no obvious autophagy induction in CRC cells (Supplementary Fig. 3G), again suggesting that OSI-induced autophagy induction might represent an EGFR-independent mechanism.

**Fig. 3:**
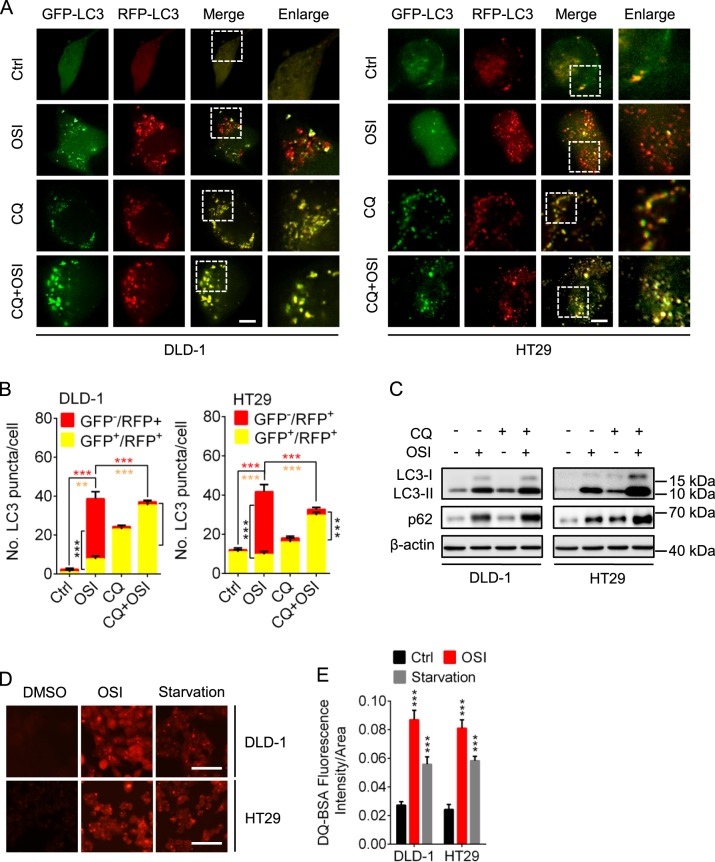
OSI promotes autophagic flux in CRC cells. **a** Immunofluorescence analysis of cells transiently transfected with tandem mRFP-GFP-tagged LC3 and treated with or without 5 μM OSI in the presence or absence of 10 μM CQ for 24 h. Scale bar, 10 μm. **b** Quantification of the ratio of red puncta indicating autolysosome (AL) versus yellow puncta indicating autophagosome (AP) per cell in (**a**). **c** CRC cells were treated with or without 5 μM OSI in the presence or absence of 10 μM CQ for 24 h. LC3 expression was examined by immunoblotting. **d** Representative images of CRC cells incubated with BODIPY-conjugated bovine serum (DQ-BSA, red) for 1 h and followed by 5 μM OSI treatment for 24 h, or incubation with serum- and glucose-free medium (Starvation). Scale bar, 20 μm. **e** The intensity of fluorescent signal in (**d**). Data are presented as mean SEM, Student’s *t*-test, and are representative of three independent experiments. **P *<* 0.01; ***P *<* 0.001

To further determine whether OSI promotes autophagic flux, we used a tandem mRFP-GFP tagged LC3 construct and found that OSI-treated CRC cells displayed increased formation of red fluorescent autolysosomes (GFP^−^RFP^+^ signal), while combinatorial treatment of CQ (chloroquine) with OSI resulted in accumulation of yellow fluorescent autophagosomes (GFP^+^RFP^+^ signal) (Fig. 3a, b). We also detected the colocalization of LC3 (autophagosome marker) with LAMP1 (lysosome marker) in OSI-treated CRC cells. As shown in Supplementary Fig. 4A, B, OSI treatment resulted in obvious colocalization of LC3 and LAMP1, suggesting that OSI promotes the fusion of the autophagosome with lysosome. In addition, combination treatment OSI with CQ led to increased accumulation of LC3-II and endogenous LC3 puncta (Fig. 3c, Supplementary Fig. 4C, D). In line with these data, using a highly self-quenched BODIPY conjugated bovine serum albumin (DQ-BSA), we found that OSI treatment increased the DQ-BSA fluorescent signal (Fig. 3d, e), suggesting increased proteolytic degradation in response to OSI treatment. Consistently, ubiquitinated protein conjugates were decreased following OSI treatment (Supplementary Fig. 4E, F). Taken together, these findings demonstrate that OSI promotes autophagic flux in CRC cells. We also analyzed the protein levels of p62/SQSTM1, which is supposed to be downregulated during a complete autophagy flux due to its degradation in autolysosome^26^. Unexpectedly, the protein levels of p62/SQSTM1 were upregulated after OSI treatment (Fig. 2a), which might contribute to the enhanced transcription of p62/SQSTM1 induced by OSI (Supplementary Fig. 5).

### Inhibition of autophagy augments the antitumor effect of OSI in CRC cells

**Fig. 4:**
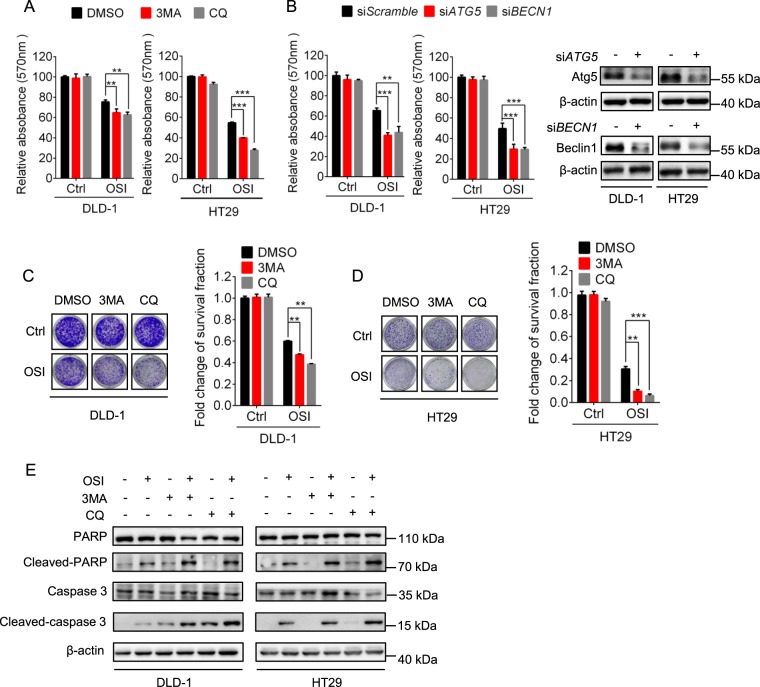
Inhibition of autophagy exacerbates OSI-induced growth suppression of CRC cells. **a** The relative absorbance at 570 nm of CRC cells treated with 5 μM OSI in the absence or presence of 10 μM CQ or 5 mM 3-MA for 24 h. **b** The relative absorbance at 570 nm of CRC cells transfected with si*Scramble*, si*ATG5*, or si*BECN1* for 24 h, followed by treatment with or without 5 μM OSI for another 24 h. **c** DLD-1 cells were treated with 10 μM CQ or 5 mM 3-MA in the presence or absence of 5 μM OSI for 24 h, followed by crystal violets staining and quantification of survival fraction. (Left) Representative images of colony formation assays of DLD-1 cells, (Right) quantification of survival fraction. **d** HT29 cells were treated with 10 μM CQ or 5 mM 3-MA in the presence or absence of 5 μM OSI for 24 h, followed by crystal violets staining and quantification of survival fraction. (Left) Representative images of colony formation assays of DLD-1 cells, (Right) quantification of survival fraction. **e** CRC cells were treated with or without 5 μM OSI in the presence or absence of 10 μM CQ or 5 mM 3-MA for 24 h. cleaved-PARP and cleaved-caspase 3 were examined by immunoblotting. Data are presented as mean SEM, Student’s *t*-test, *P < 0.05; **P *<* 0.01; ***P *<* 0.001

To evaluate whether autophagy was involved in the anti-CRC effect of OSI, CRC cells were treated with OSI combined with 3-MA, si*ATG5*, si*BECN1*, or CQ, respectively. As shown, combinational use of autophagy inhibitors (3-MA or CQ), si*ATG5* or si*BECN1* with OSI significantly potentiated OSI-induced growth suppression in CRC cells (Fig. 4a–d). In line with these observations, OSI-induced apoptotic cell death was augmented in the presence of autophagy inhibitors, as indicated by increased levels of cleaved-PARP and cleaved-caspase 3 (Fig. 4e). Collectively, these data demonstrate that inhibition of autophagy significantly promotes OSI-induced CRC suppression, partially by enhancing apoptosis induction, suggesting a protective role of autophagy in OSI-treated CRC cells.

### LKB1/AMPK signaling plays a major role in OSI-induced autophagy

**Fig. 5:**
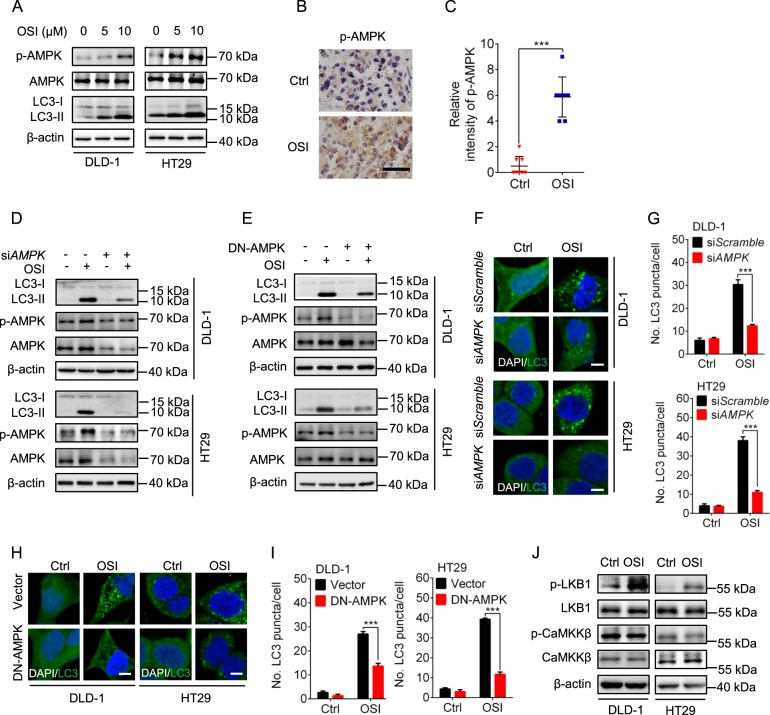
OSI induces autophagy by activating LKB1/AMPK signaling in CRC cells. **a** Immunoblotting analysis of AMPK, phosphorylated AMPK (Thr172) and LC3 levels in CRC cells treated with indicated concentration of OSI for 24 h. **b** Immunohistochemistry analysis of AMPK phosphorylation levels in xenograft tissues. Scale bar, 50 μm. **c** Relative intensity of phosphorylated AMPK staining in (**b**). **d** CRC cells were transfected with si*Scramble* or si*AMPK* for 24 h and followed by treatment with or without 5 μM for another 24 h. LC3 and phosphorylated AMPK (Thr172) levels were detected by immunoblotting. **e** CRC cells were transfected with empty vector or DN-AMPK plasmid for 24 h, followed by treatment with or without 5 μM OSI for another 24 h. LC3 and AMPK phosphorylation levels were determined by immunoblotting. **f** CRC cells were treated as in (**d**), the endogenous LC3 puncta in CRC cells were assessed by immunofluorescence. Scale bar, 10 μm. **g** The number of LC3 puncta per cell in (**f**). **h** CRC cells were treated as in (**e**), the endogenous LC3 puncta in CRC cells were assessed by immunofluorescence, Scale bar, 10 μm. **i** The number of LC3 puncta per cell in (**h**). **j** Immunoblotting analysis of phosphorylated LKB1 and phosphorylated CaMKKβ levels in CRC cells treated with or without 5 μM OSI. Data are presented as mean SEM, Student’s *t*-test, and are representative of three independent experiments. ***P *<* 0.001

It has been previously reported that AMPK is a canonical upstream signaling molecule for autophagy induction^27^. Therefore, we investigated the phosphorylation status of AMPK to validate whether AMPK was involved in OSI-induced autophagy^28,29^. As shown in Fig. 5a, the phosphorylation levels of AMPK were enhanced after OSI treatment. In addition, OSI treatment resulted in relatively stronger staining of phosphorylated AMPK (Thr172) in CRC xenografts treated with OSI compared with the control group (Fig. 5b, c). Inactivation of AMPK, either by siRNA or a dominant-negative mutant of AMPK (DN-AMPK), significantly inhibited OSI-induced LC3-II conversion (Fig. 5d, e) and LC3 puncta accumulation (Fig. 5f–i). We then examined whether LKB1 or CaMKKβ, two well-known AMPK upstream kinases, were responsible for the activation of AMPK in OSI-treated CRC cells. As shown in Fig. 5j, the phosphorylation level of LKB1, but not CaMKKβ, was enhanced following OSI treatment. Consistently, siRNA-mediated LKB1 silencing markedly inhibited OSI-induced LC3-II conversion (Supplementary Fig. 6A). These results suggest that OSI induces autophagy by activating LKB/AMPK signaling pathway in CRC cells.

### OSI induces autophagy through MCT1-mediated activation of AMPK in CRC cells

**Fig. 6:**
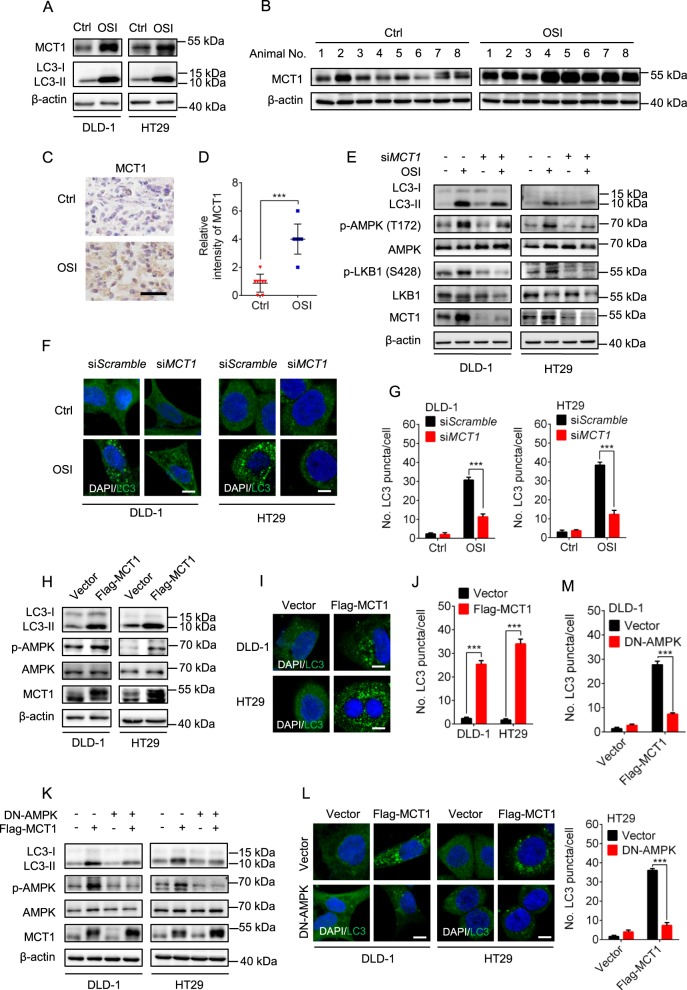
OSI induces autophagy through upregulation of MCT1 in CRC cells. **a** Immunoblotting analysis of MCT1 expression in CRC cells treated with or without 5 μM OSI for 24 h. **b** Immunoblotting analysis of MCT1 and phosphorylated AMPK in tumor xenografts obtained from vehicle- or OSI-treated mice. (Each protein of interest from each group was electrophoretically transferred onto a PVDF membrane, incubated with indicated primary and secondary antibodies, and developed as a digital image.) **c** Immunohistochemical analysis of MCT1 expression in tumor xenografts. Scale bar, 50 μm. **d** Relative intensity of MCT1 staining in (**c**). **e** CRC cells were transfected with si*Scramble* or si*MCT1* for 24 h, followed by treatment with or without 5 μM OSI for another 24 h. The protein levels of LC3, phosphorylated AMPK, phosphorylated LKB1 and MCT1 were analyzed by immunoblotting. **f** CRC cells were treated as in (**e**), the endogenous LC3 puncta were analyzed by immunofluorescence. Scale bar, 10 μm. **g** The number of LC3 puncta in (**f**). **h** CRC cells were transfected with empty vector or Flag-MCT1 plasmid for 48 h, the protein levels of MCT1 and phosphorylated AMPK were analyzed by immunoblotting. **i** CRC cells were treated as in (**h**), the endogenous LC3 puncta were analyzed by immunofluorescence. Scale bar, 10 μm. **j** The number of LC3 puncta in (**i**). **k** Immunoblotting analysis of LC3, MCT1 and phosphorylated AMPK levels in CRC cells co-transfected with Flag-MCT1 and DN-AMPK plasmids for 48 h. **l** CRC cells were treated as in (**k**), the endogenous LC3 puncta were analyzed by immunofluorescence. Scale bar, 10 μm. **m** The number of LC3 puncta per cell in (**l**). Data are presented as mean SEM, Student’s *t*-test, and are representative of three independent experiments. *P < 0.05; ***P *<* 0.001

The lactate transporter MCT1 has been previously reported to be associated with AMPK activation^30^. We thus speculated that MCT1 might be involved in AMPK-mediated autophagy induction in OSI-treated CRC cells. We found that OSI treatment led to upregulation of MCT1 in CRC cells (Fig. 6a), but olmutinib failed to elevate the protein level of MCT1 (Supplementary Fig. 3G). The upregulation of MCT1 in response to OSI treatment was further confirmed in xenograft tumors (Fig. 6b–d). Next, we investigated whether MCT1 was required for OSI-induced AMPK activation and autophagy induction in CRC cells by knockdown or enforced expression of MCT1. As shown in Fig. 6e–g, siRNA-mediated *MCT1* silencing markedly repressed the OSI-induced phosphorylation levels of LKB1 and AMPK, LC3-II conversion and LC3 puncta accumulation. In addition, exogenous MCT1 expression resulted in elevated LC3-II conversion (Fig. 6h), LC3 puncta accumulation (Fig. 6i, j) and AMPK phosphorylation, to a similar level to that observed in cells treated by OSI alone. Notably, the increased LC3 lipidation induced by MCT1 overexpression could be counteracted by DN-AMPK-mediated AMPK inactivation (Fig. 6k–m) or *LKB1* knockdown (Supplementary Fig. 6B). Taken together, these results demonstrate that OSI upregulates the expression of MCT1, which leads to the activation of the LKB1/AMPK signaling pathway, thereby inducing autophagy in CRC cells.

### MCT1 antagonizes the antitumor efficacy of OSI and is overexpressed in human CRC tissues

**Fig. 7:**
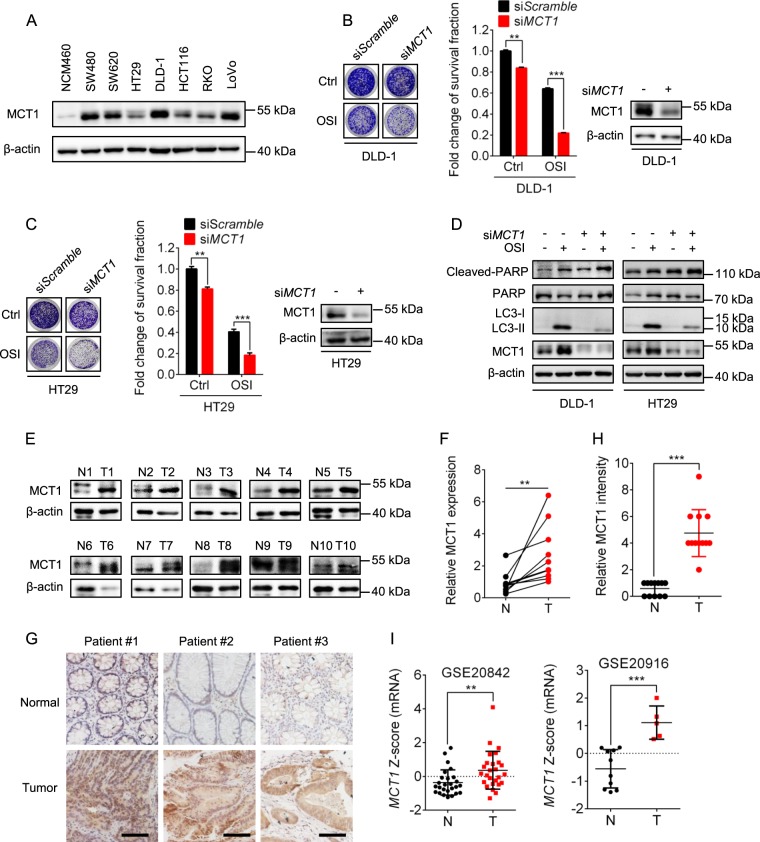
MCT1 antagonizes the antitumor efficacy of OSI and is overexpressed in human CRC tissues. **a** Immunoblotting analysis of MCT1 protein levels in CRC cell lines and noncancerous colorectal cell line NCM460. **b**, **c** Representative images of colony formation assays of CRC cells transfected with si*Scramble* or si*MCT1* for 24 h, followed by treatment with or without 5 μM OSI for another 24 h. **d** Immunoblotting analysis of cleaved-PARP expression in CRC cells transfected with si*Scramble* or si*MCT1* for 24 h, followed by treatment with or without 5 μM OSI for another 24 h. **e** Immunoblotting analysis of MCT1 expression in 10 pairs of tumor and adjacent normal tissues obtained from CRC patients. **f** Relative quantification of MCT1 levels in (**e**). **g** Immunohistochemical staining of MCT1 in 10 pairs of tumor and adjacent normal tissues obtained from CRC patients. Scale bar, 50 μm. **h** Relative intensity of MCT1 levels in (**g**). **i** Data represent the *Z*-score of MCT1 from Oncomine database. Data are presented as mean SEM, Student’s *t*-test, and are representative of 3 independent experiments. **P < 0.01; ***P *<* 0.001

Given the role of MCT1 in mediating OSI-induced autophagy, it was necessary to explore whether MCT1 interfered with the OSI-induced CRC suppression. As shown in Fig. 7a, MCT1 was overexpressed in various CRC cells compared with noncancerous NCM460 cells. siRNA-mediated *MCT1* silencing remarkably aggravated the OSI-induced growth suppression in CRC cells (Fig. 7b, c), at least in part, by promoting apoptosis as evidenced by the enhanced levels of cleaved- PARP (Fig. 7d). Interestingly, there was no synergistic effect on CRC suppression and LC3-II conversion of OSI with AZD3965 (a MCT1 inhibitor that blocks its lactate transporting function) (Supplementary Fig. 7A, C), implying that the mechanism of MCT1-mediated autophagy might be independent of the monocarboxylate-transporting function of MCT1.

As OSI has been reported to induce autophagy in NSCLC cells^31^, we analyzed whether MCT1 and protective autophagy were involved in the resistance of NSCLC cells to OSI. We found that the protein level of MCT1 was upregulated after OSI treatment both in T790M mutation harboring NCI-1975 cells and EGFR WT A549 cells (Supplementary Fig. 8A, C). Moreover, silencing MCT1 augmented OSI-induced growth suppression in NSCLC cells (Supplementary Fig. 8B, D). These results indicate that the upregulation of MCT1 and subsequent autophagy initiation might play a role in the drug resistance of NSCLC cells to OSI.

It has been reported that MCT1 is overexpressed in many cancers, such as HCC and gastric cancer^16^. Here, our study suggested that the expression of MCT1 was upregulated in CRC specimens compared with normal tissues by immunoblot and immunohistochemical analysis (Fig. 7e–h), which was consistent with the Oncomine data (Fig. 7i). These data indicate that MCT1 may play important role in CRC cell growth. Together, our study suggests that MCT1 might confer resistance to the antitumor effect of OSI, and may serve as a promising therapeutic target for CRC.

### OSI enhances the anti-CRC efficacy of 5-FU and oxaliplatin

5-Fluorouracil and oxaliplatin, two first-line chemotherapeutic drugs for clinical CRC treatment, have been reported to lead to drug resistance^32,33^. A recent study demonstrated that OSI increases sensitivity of NSCLC cells to radiation by delaying DNA damage repair^34^. Thus, we question whether OSI could also enhance the chemosensitivity of CRC cells to 5-FU or oxaliplatin. As indicated, combinational treatment of OSI with 5-FU or oxaliplatin markedly decreased the relative absorbance (Supplementary Fig. 9A, 9B) and proliferation rate (Supplementary Fig. 9C, 9D) of CRC cells. Taken together, these results demonstrate that OSI effectively sensitizes CRC cells to 5-FU and oxaliplatin treatment, which was consistent with a previous study suggesting that OSI synergizes with 5-FU^35^, and the underlying molecular mechanism remains rarely unexplored.

## Discussion

OSI is a first-line chemotherapeutic agent for NSCLC patients harboring the EGFR T790M mutation^36,37^. Interestingly, Hirano et al. suggested that OSI has potent efficacy against NSCLC harboring EGFR 19 deletion and L858R mutation, as well as those harboring exon 20 insertion mutations, suggesting a wide therapeutic window of OSI in NSCLC^38^. In this study, we found that OSI has potent anticancer effects in CRC, which might interestingly be independent of its selective inhibitory activity targeting EGFR T790M mutation. Our findings may extend the clinical potential of OSI and provide a new paradigm for cancer therapy.

Autophagy was proposed as a potential cell death mechanism during ionizing radiation and chemotherapy^39^. However, accumulating evidence suggests that autophagy could facilitate the resistance of cancer cells to chemotherapeutic drugs, and inhibition of autophagy could sensitize tumor cells to cancer therapies. Thus, it is necessary to determine the role of autophagy in cancer therapy for rational manipulation of autophagy to assist chemotherapy. Our data demonstrate that OSI induces autophagic flux, which counteracts apoptosis induction and growth suppression of CRC cells. Hence, it is reasonable to conclude that autophagy plays a protective role against OSI-induced CRC suppression. It has been reported that OSI could induce acquired drug resistance in NSCLC patients^40^, mainly due to a second mutation of EGFR (such as C797 mutation)^41^, or activation of by-pass pro-survival signaling pathways such as MET/ERK, HER2, IRE1α or RAS^41–44^. Our data demonstrate autophagy as an additional drug resistance mechanism for OSI treatment in both NSCLC and CRC, suggesting that combinatorial use of OSI with autophagy inhibitors may benefit their therapeutic efficacy for cancer treatment. Further studies are needed to detect the combinatorial efficacy of OSI and autophagy inhibitors in vivo.

AMPK, a well-known energy sensor, could switch on glycolysis and induce autophagy to maintain ATP level^45^. It has been reported that AMPK could phosphorylate ULK1 to form PI3K complex, leading to autophagy induction^46,47^. Our study reveals that LKB1 mediated-activation of AMPK is crucial for the initiation of autophagy in OSI-treated CRC cells. Further investigation shows that OSI treatment upregulates the protein level of MCT1, which subsequently activates LKB1/AMPK signaling, leading to autophagy induction in CRC cells. The MCT1/LKB1/AMPK is proposed as a new mechanism of autophagy modulation axis. A previous study has shown that AMPK could be activated by MCT1-mediated lactate uptake^30^. Further studies are needed to delineate the mechanism underlying MCT1-mediated activation of LKB1/AMPK signaling and subsequent autophagy induction.

MCT1 plays an important role in metabolic reprogramming by transporting of monocarboxylates such as lactate, pyruvate, and ketone bodies from hypoxic cancer cells or cancer-associated fibroblasts (CAFs) to aerobic cancer cells^48^. Robust lactate metabolism in MCT1-positive cancer cells produces sufficient energy for self-renewal and distant metastasis, indicating a predominant role of MCT1-positive cancer cells in tumor environment^12,49^. Here, we found that MCT1 is overexpressed in CRC tissue and cell lines. Importantly, MCT1 is upregulated to activate the LKB1/AMPK signaling pathway and subsequently initiates protective autophagy in OSI-treated CRC cells, conferring resistance to OSI. Our findings together with previous studies indicate that intervening MCT1 under OSI treatment could improve treatment efficacy and outcome in CRC tumors^50^. However, our further data suggests that MCT1 knockdown, rather than pharmacological inhibition of its lactate transporting function by AZD3965, suppresses autophagy induction and aggravates the antitumor effect of OSI in CRC cells. This could be attributed to MCT4 heterogeneous expression as AZD3965 has been reported to kill preferentially tumor cells with high MCT1 and low MCT4 expression^51^. In addition, the role of MCT1 in modulating autophagy might be in a non-canonical role of MCT1, independent of its lactate transporter activity. Indeed, MCT1 has been reported to activate the transcription factor NF-κB to promote tumor metastasis, beyond its role as a lactate transporter^14^. Further studies should be conducted to investigate the efficacy of combinatorial use of OSI with MCT1 inhibitors in vivo.

In conclusion, we have demonstrated that OSI has a pronounced anti-CRC effect both in vitro and in vivo by stimulating apoptosis. Meanwhile, OSI upregulates the expression of MCT1 in CRC cells, which in turn promotes the induction of protective autophagy (Supplementary Fig. 10). Our study indicates that MCT1 might be an attractive therapeutic target for CRC treatment, and reveals the potency of OSI as a promising therapeutic agent for CRC treatment.

## Supplementary information

supplementary figure and supplementary figure legends
