# Supplementary material for: MCT1 relieves osimertinib-induced CRC suppression by promoting autophagy through the LKB1/AMPK signaling
Source: Cell Death Dis. 2019 Aug 13;10(8):615. doi: 10.1038/s41419-019-1844-2 (PMC6692318; doi:10.1038/s41419-019-1844-2)

Supplementary Figure Legends

Supplementary Figure 1. OSI holds potential inhibitory effect on CRC cell growth.

(A) Quantification of relative inhibitory rates of different CRC cells treated with 15 EGFR inhibitors for 48 hours. (B) The concentrations of 15 EGFR inhibitors used in (A). (C) The KRSA status of the different cell lines included in this work. (D) The EGFR expression level of the different cell lines included in this work.

Supplementary Figure 2. OSI exhibits potent anti-CRC effect and has no obvious systemic toxicity in mice.

(A) Representative image of isolated tumors of mice in cohorts treated with vehicle (n=8) or OSI (n=8) (15 mg/kg/day). Treatment was initiated 24 hours after tumor reaching ~200 mm^3. (B) The weight of individual tumors in (A). (C) H&E staining of the intestine, heart, liver, lung, spleen, and kidney in mice treated with vehicle or OSI (15 mg/kg/day). Scale bar, 50 μm. (D) Body weights of mice measured at the indicated time points. Data are presented as mean SEM, Student's t-test, and are representative of 3 independent experiments.

Supplementary Figure 3. OSI, but not olmutinib, triggers autophagy in CRC cells.

(A) Immunoblotting analysis of LC3 turnover in DLD-1 and HT29 cells treated with 5 μM OSI for indicated times. (B) Immunoblotting analysis of LC3 turnover in HCT116,

SW480, SW620 cells treated with or without 5 μM OSI for 24 hours. (C) Immunofluorescence analysis of LC3 puncta in CRC cells transfected with empty vector or GFP-LC3 plasmid for 24 hours and followed by treatment with or without 5 μM OSI for another 24 hours. (D) Total number of LC3 puncta per cell in (C). (E-F) Total number of LC3 puncta per cell in Fig. 2J and Fig. 2M. (G) Immunoblotting analysis of the protein levels of MCT1, Atg5 and LC3 turnover in DLD-1 and HT29 cells treated with indicated concentration of olmutinib for 24 hours. Data are presented as mean SEM, Student's t-test, and are representative of 3 independent experiments. ***, $P < 0.001$.

Supplementary Figure 4. OSI promotes the autolysosome degradation in CRC cells.

(A) Immunofluorescence analysis of the co-localization of endogenous LC3 and LAMP1 in DLD-1 and HT29 cells treated with or without 5 μM OSI for 24 hours. Scale bar, 10 μM. (B) The number of co-localized puncta of LC3 and LAMP1 per cell in (A). (C) Immunofluorescence analysis of endogenous LC3 puncta in CRC cells treated with or without 5 μM OSI in the presence or absence of 10 μM CQ. (D) Total number of LC3 puncta per cell in (A). (E) Immunoblotting analysis of LC3 and ubiquitinated protein levels in CRC cells treated with indicated concentrations of OSI for 24 hours. (F) Time course analysis of ubiquitinated protein levels and LC3 turnover by immunoblotting in DLD-1 and HT29 cells treated with 5 μM OSI for indicated times.

Data are presented as mean SEM, Student's t-test, and are representative of 3 independent experiments. ***, $P < 0.001$.

Supplementary Figure 5. OSI enhances the expression of p62/SQSTM in CRC cells.

(A) CRC cells were treated with or without 5 μM OSI in the presence or absence of 100 μg/ml cycloheximide (CHX) 24h, p62/SQSTM were examined by immunoblotting to evaluate its degradation rate. (B) CRC cells were treated with or without 5 μM OSI in the presence or absence of 10 μM MG132 for 10h before they were harvested, p62/SQSTM were examined by immunoblotting to evaluate its translation ability. (C) CRC cells were treated with indicated concentration of OSI for 24h, Q-PCR analysis was performed to assess the mRNA level of p62/SQSTM. Data are presented as mean SEM, Student's t-test, *, $P < 0.05$; **, $P < 0.01$; ***, $P < 0.001$.

Supplementary Figure 6. LKB1 is involved in MCT1–mediated autophagy initiation.

(A) DLD-1 cells were transfected with si*Scramble* or si*LKB1* for 24 hours, followed by treatment with or without 5 μM OSI for another 24 hours. The protein levels of LC3, phosphorylated AMPK and phosphorylated LKB1 were analyzed by immunoblotting. (B) Immunoblotting analysis of LC3, phosphorylated AMPK, phosphorylated LKB1 and MCT1 levels in DLD-1 cells cotransfected with Flag-MCT1 and si*LKB1* for 48

hours.

Supplementary Figure 7. MCT1 inhibitor AZD3965 has no obvious effect on OSI-mediated CRC suppression.

(A) Representative images of colony formation assays of CRC cells treated with or without 5 μM OSI in the presence or absence of AZD3965 (10 μM) for 24 hours. (B) Relative absorbance at 570 nm of CRC cells treated with or without 5 μM OSI in the presence or absence of AZD3965 (10 μM) for 24 hours. (C) Immunoblotting analysis of LC3 turnover in CRC cells treated with or without 5 μM OSI in the presence or absence of AZD3965 (10 μM) for 24 hours.

Supplementary Figure 8. Silencing of MCT1 exaggerates the antitumor efficacy of OSI in human NSCLC.

(A) Immunoblotting analysis of MCT1 expression in human NCI-H1975 cells treated with or without 5 μM OSI for 24 hours. (B) (Middle) Representative images of colony formation assays of human NCI-H1975 cells treated with or without 5 μM OSI after transfection with si*Scramble* or si*MCT1* plasmids. (Right) human NCI-H1975 cells were treated as described in (left), followed by crystal violets staining and quantification of survival fraction. (C) Immunoblotting analysis of MCT1 expression in human A549 cells treated with or without 5 μM OSI for 24 hours. (C) (Middle) Representative images of colony formation assays of human NCI-H1975 cells treated

with or without 5 μM OSI after transfection with si*Scramble* or si*MCT1* plasmids. (Right) human A549 cells were treated as described in (left), followed by crystal violets staining and quantification of survival fraction.Data are presented as mean SEM, Student's t-test, and are representative of 3 independent experiments. **, $P < 0.01$; ***, $P<0.001$.

Supplementary Figure 9. OSI enhances the anti-CRC efficacy of 5-FU and Oxaliplatin.

(A) Relative absorbance at 570 nm of CRC cells treated with vehicle or 50 μM 5-FU in the presence or absence of OSI (1 μM) for 24h. (B) Relative absorbance of CRC cells treated with vehicle or 50 μM oxaliplatin in the presence or absence of OSI (1 μM) for 24h. (C) Representative images of colony formation assays of CRC cells treated with vehicle or 50 μM 5-FU in the presence or absence of OSI (1 μM) for 24 hours. (D) Representative images of colony formation assays of CRC cells treated with vehicle or 50 μM oxaliplatin in the presence or absence of OSI (1 μM) for 24 hours. Data are presented as mean SEM, Student's t-test, and are representative of 3 independent experiments. **, $P < 0.01$; ***, $P < 0.001$

Supplementary Figure 10. A schematic model illustrating the molecular mechanism of OSI-induced autophagy in CRC cells.

OSI upregulates the expression of MCT1 to activate the LKB1/AMPK signaling, thus

inducing protective autophagy in CRC cells. Inhibition of autophagy markedly augments OSI-induced apoptosis and growth inhibition in CRC cells.

Supplementary Figure 1. OSI holds potential inhibitory effect on CRC cell growth.

A

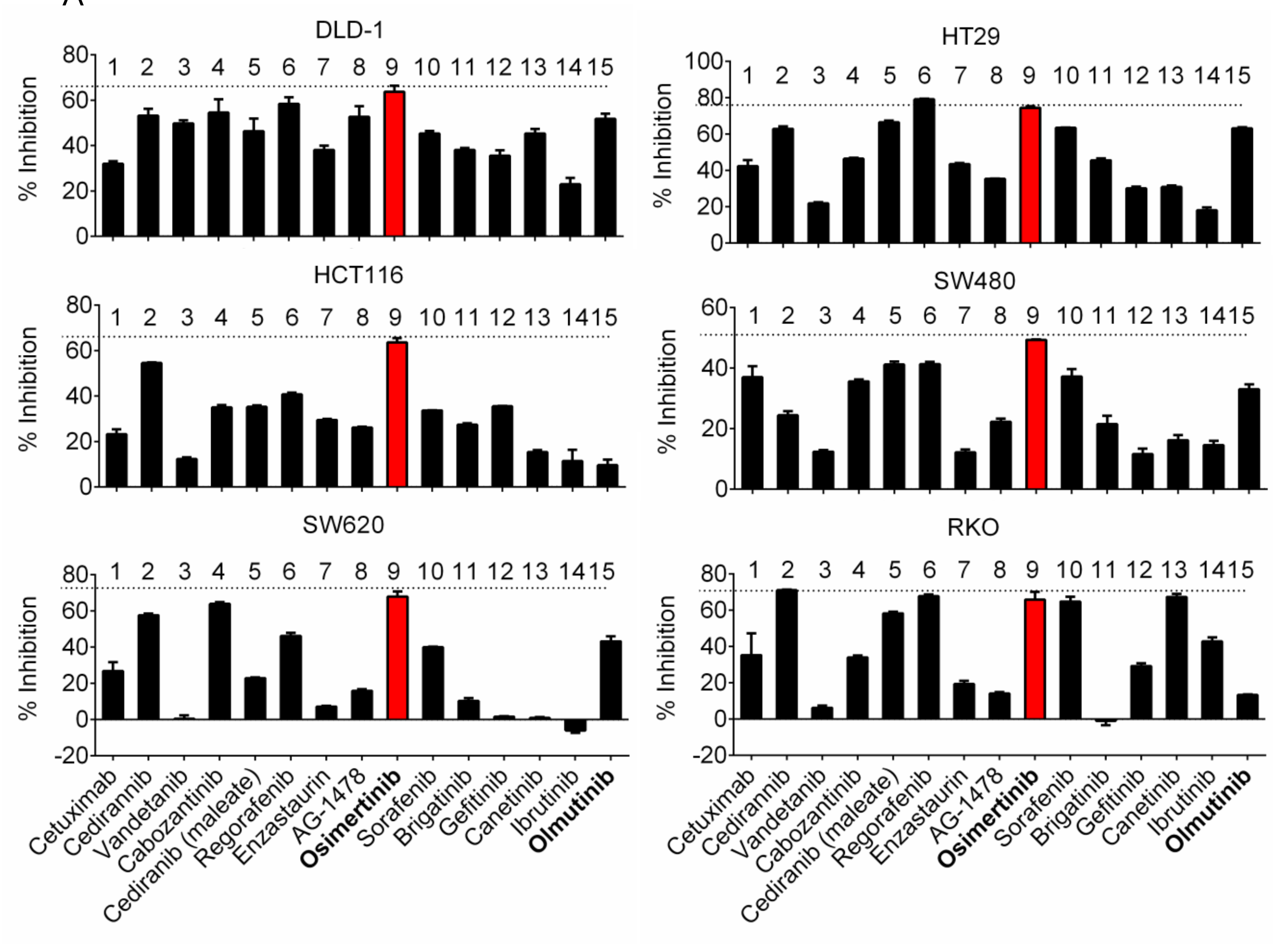

B

No.	Concentration	No.	Concentration
1	100ug/ml	**9**	5 μM
2	5 μM	10	10 μM
3	5 μM	11	5 μM
4	8 μM	12	6 μM
5	5 μM	13	6 μM
6	5 μM	14	8 μM
7	8 μM	**15**	10 μM
8	5 μM		

C

Cell lines	KRAS status
DLD-1	G13D
HT29	wt
HCT116	G13D
SW480	G12V
SW620	G12V
RKO	wt
LoVo	G13D; A14V

D

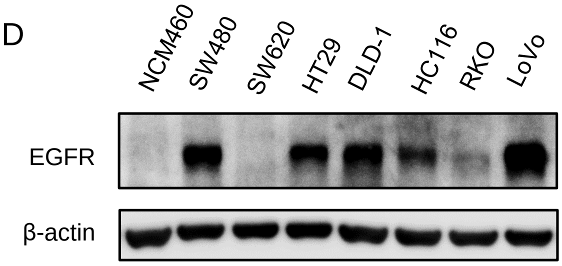

Supplementary Figure 2. OSI exhibits potent anti-CRC effect and has no obvious systemic toxicity in mice.

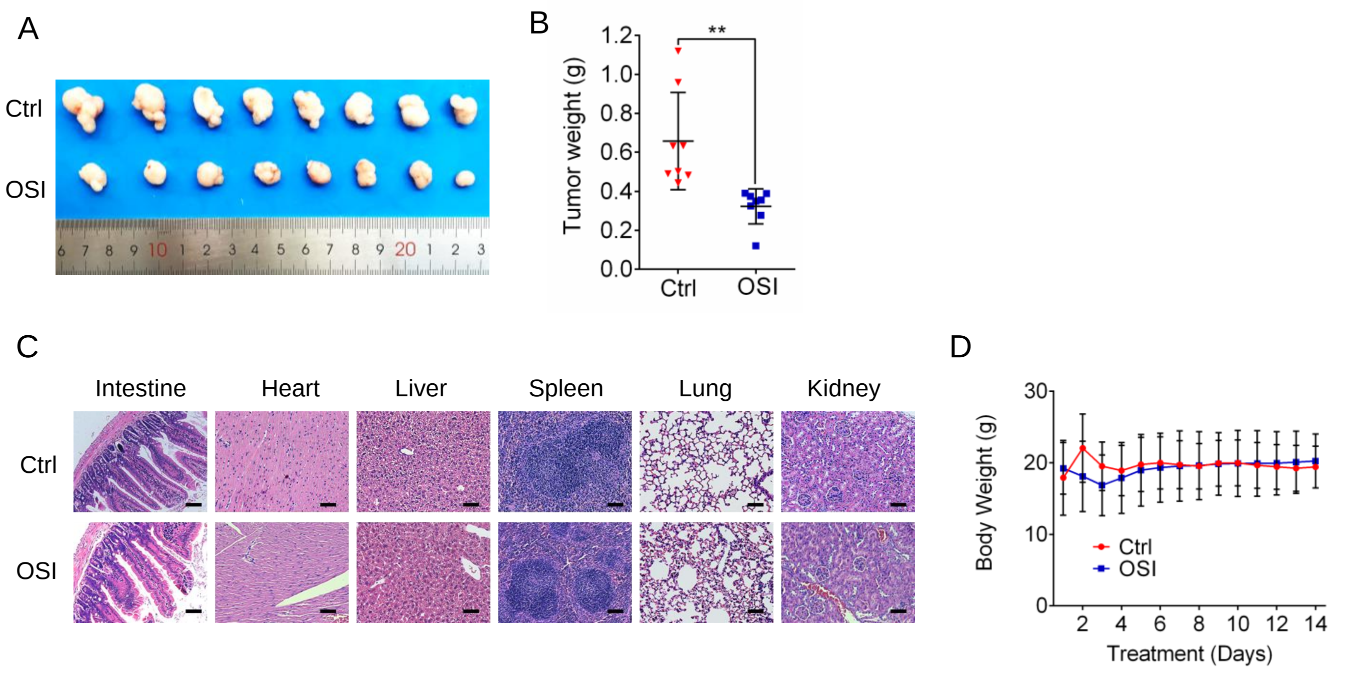

Supplementary Figure 3. OSI, but not olmutinib, triggers autophagy in CRC cells.

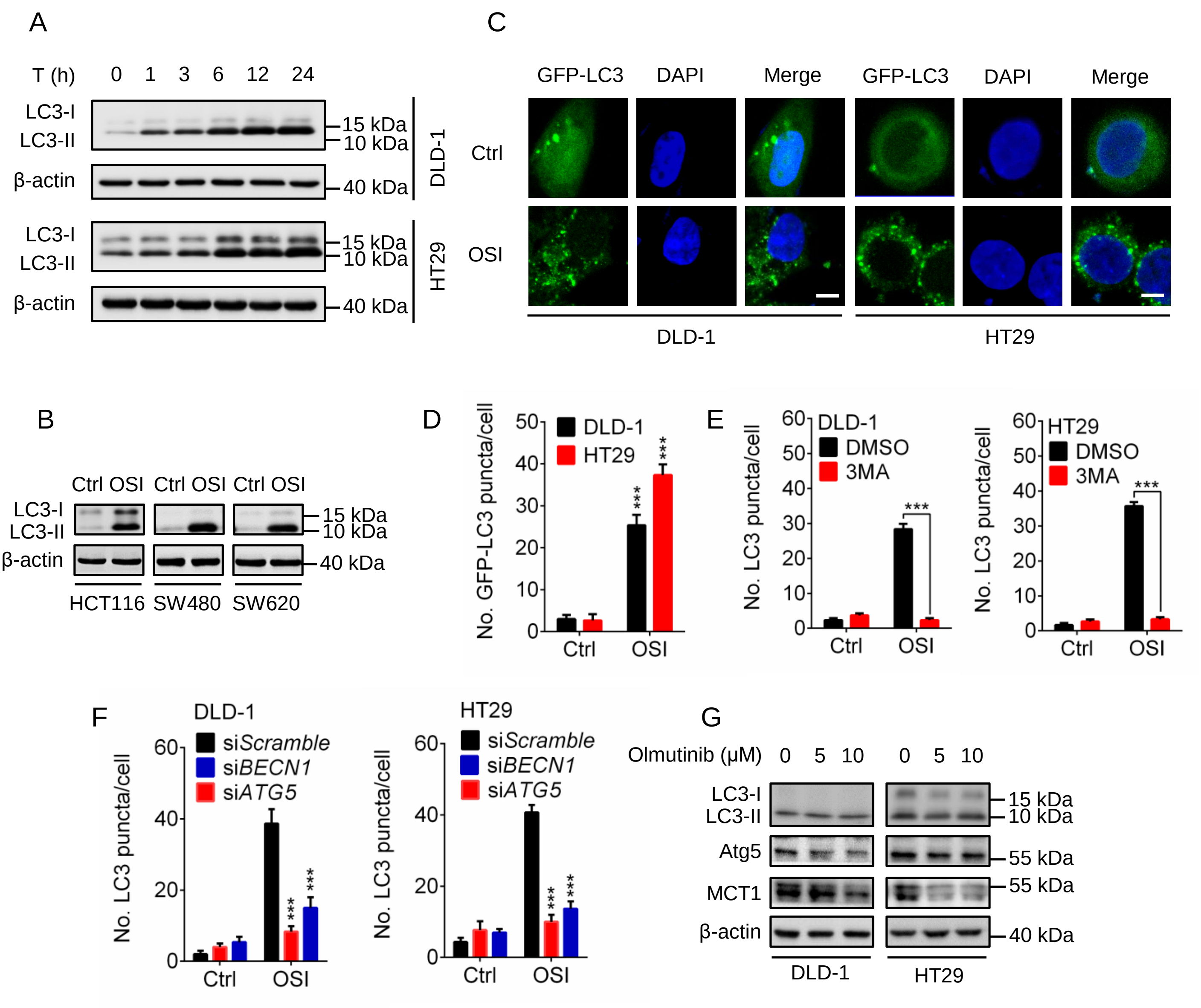

Supplementary Figure 4. OSI promotes the degradation of autolysosome in CRC cells.

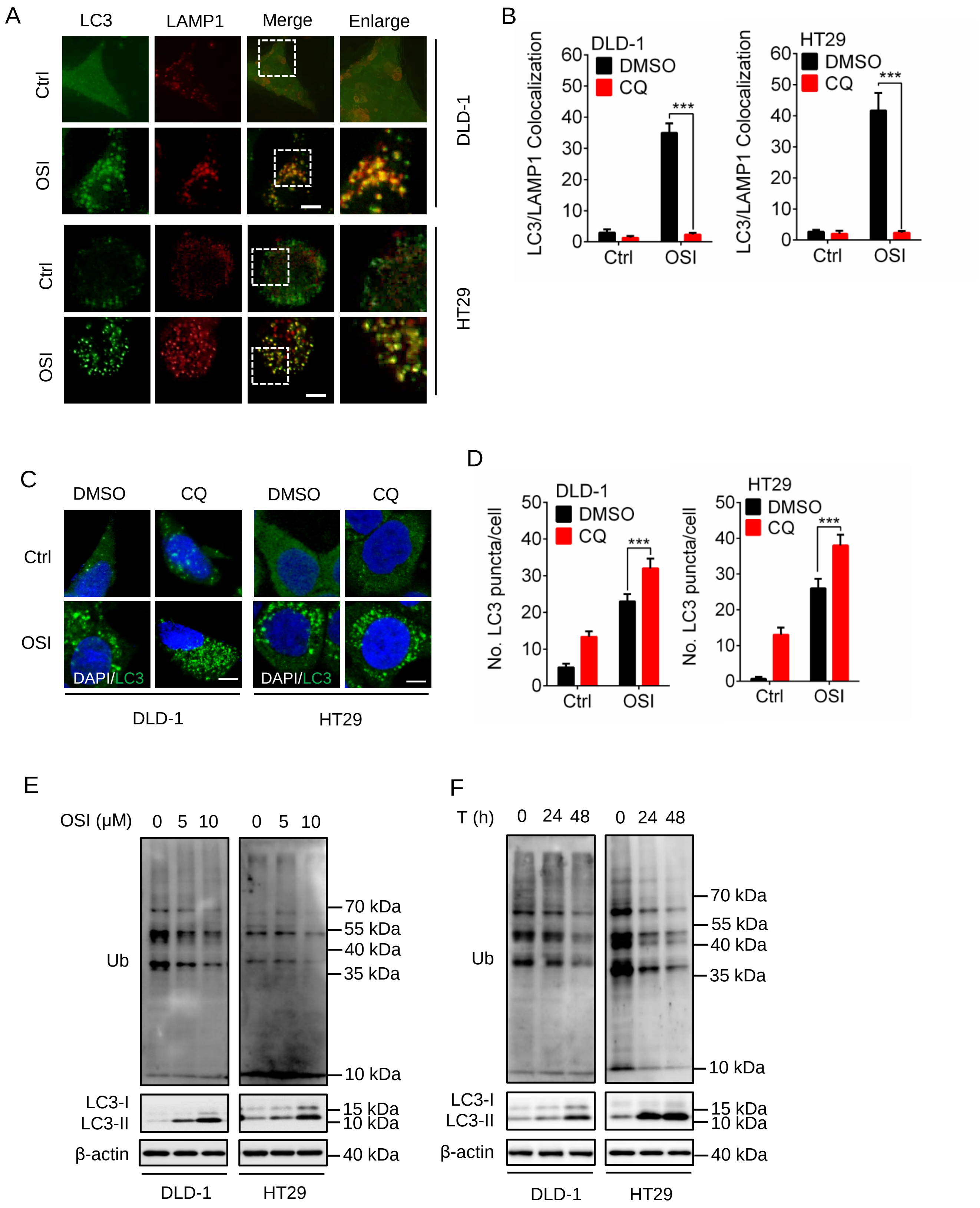

Supplementary Figure 5. OSI enhances the expression of p62/SQSTM in CRC cells

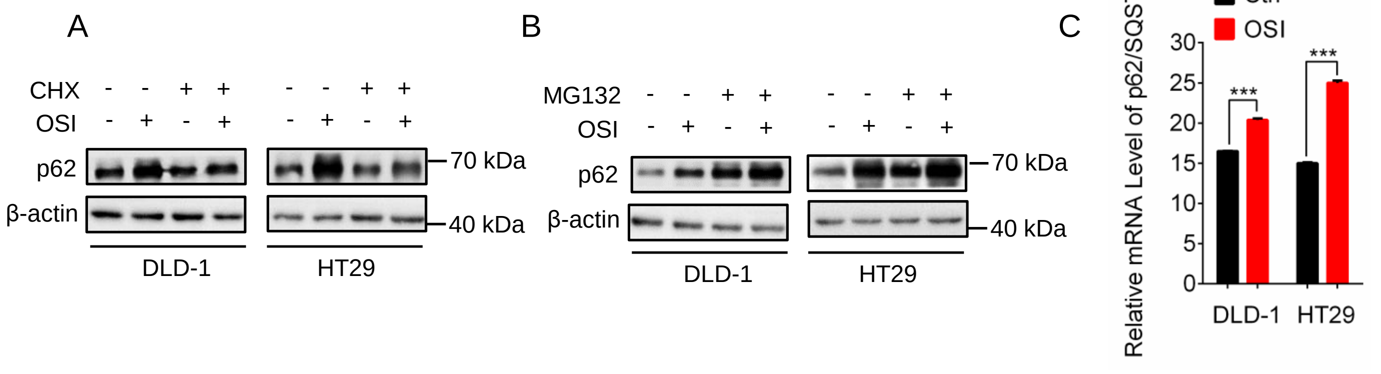

Supplementary Figure 6. LKB1 is involved in MCT1–mediated autophagy initiation.

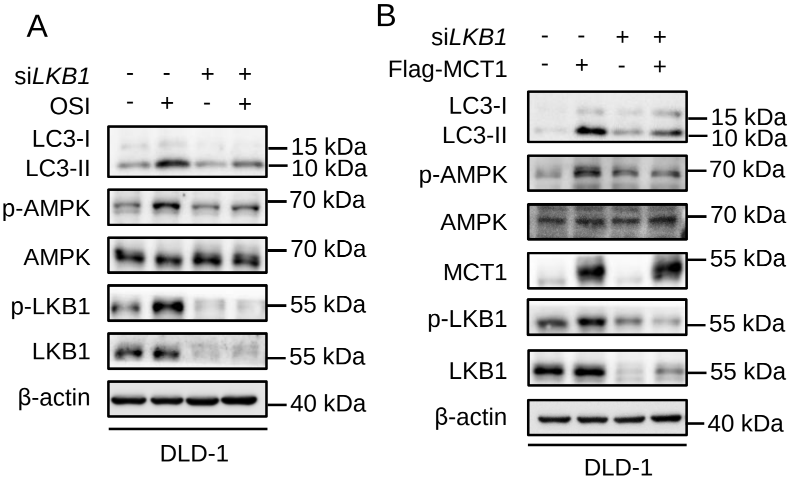

Supplementary Figure 7. MCT1 inhibitor AZD3965 has no obvious effect on the OSI mediated CRC suppression and autophagy .

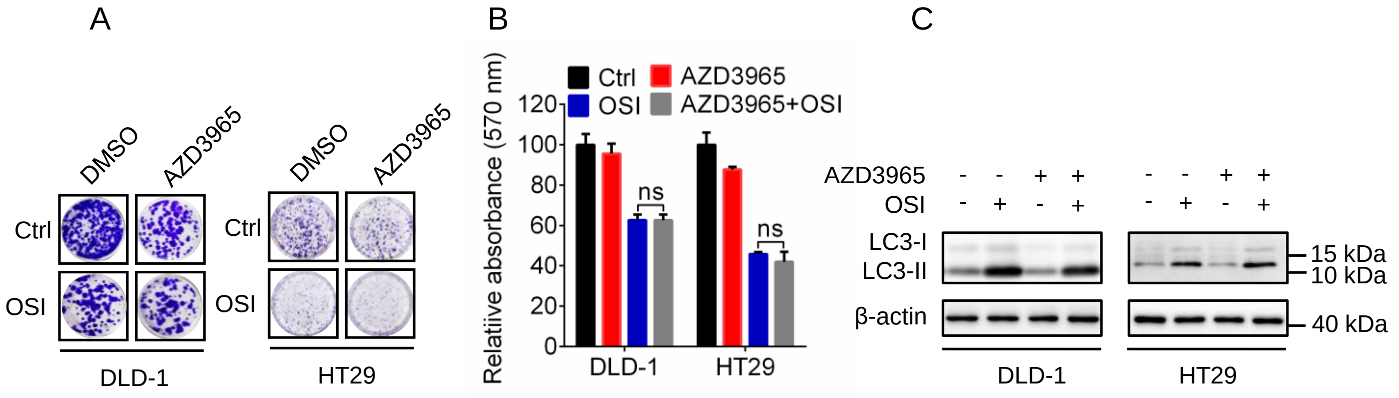

Supplementary Figure 8. MCT1 antagonize to the antitumor efficacy of OSI in human NSCLC.

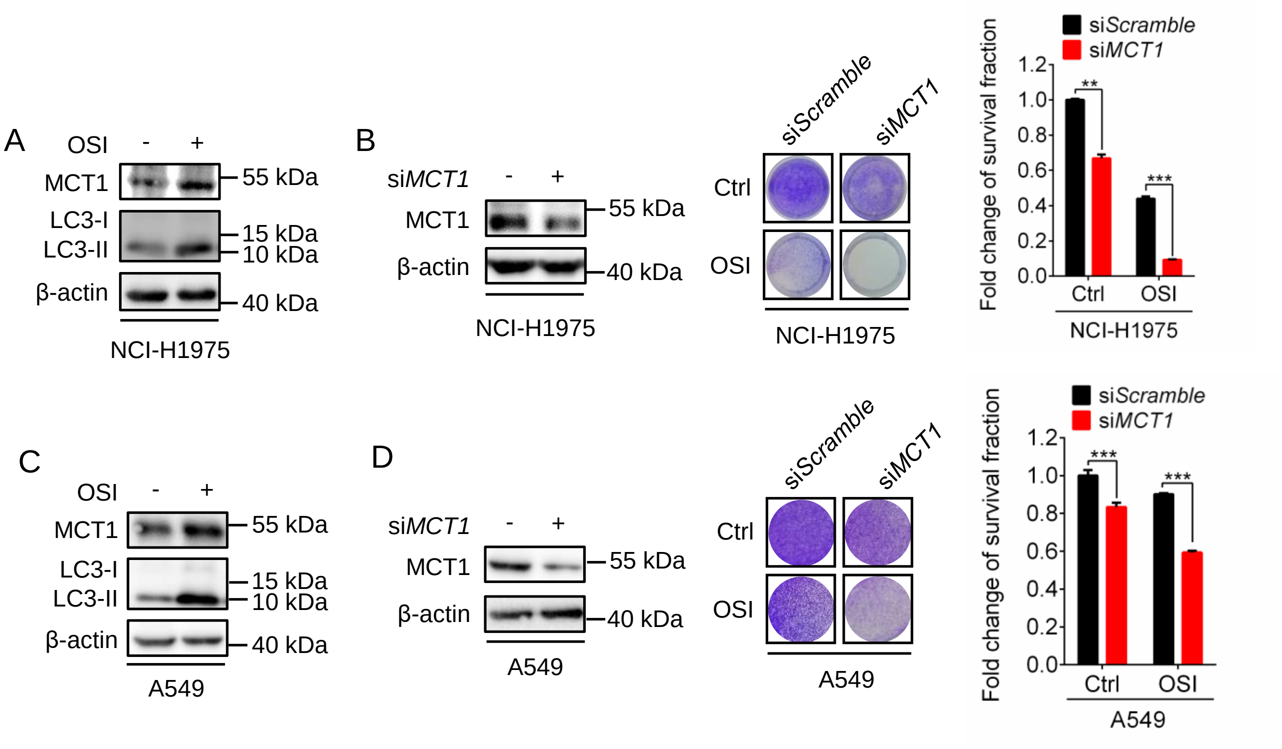

Supplementary Figure 9. OSI enhances the anti-CRC efficacy of 5-FU and Oxaliplatin.

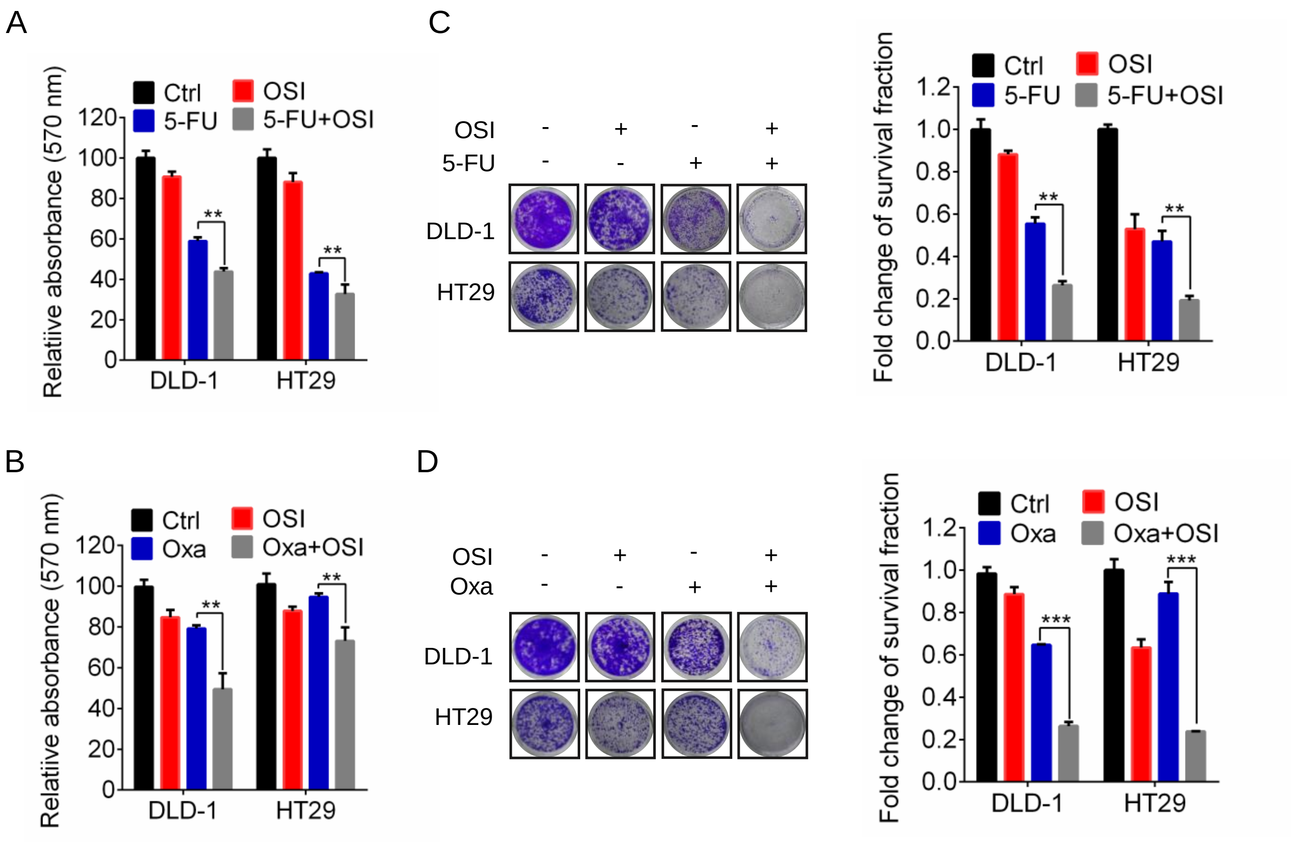

Supplementary Figure 10. A schematic model illustrating the molecular mechanism of OSI-induced autophagy in CRC cells.

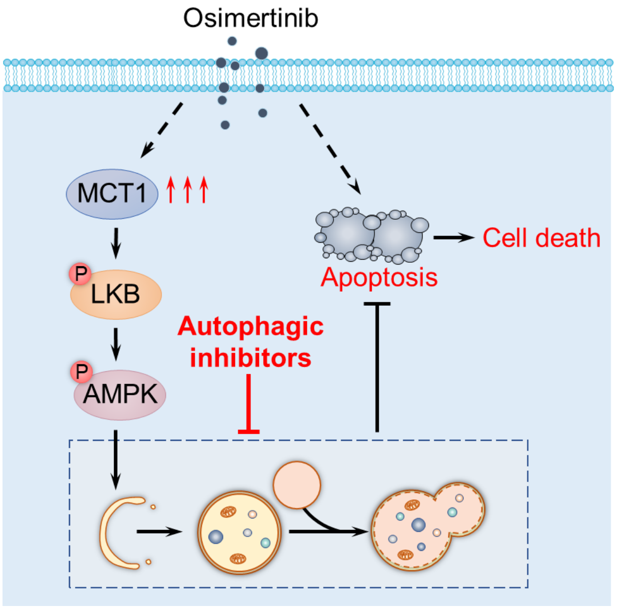

Supplement: Supplementary file 1 — supplementary figure and supplementary figure legends [file 41419_2019_1844_MOESM1_ESM.pdf]
